# The Coiled-Coil Domain of EHD2 Mediates Inhibition of LeEix2 Endocytosis and Signaling

**DOI:** 10.1371/journal.pone.0007973

**Published:** 2009-11-19

**Authors:** Maya Bar, Miya Sharfman, Silvia Schuster, Adi Avni

**Affiliations:** Department of Plant Sciences, Tel-Aviv University, Tel-Aviv, Israel; Purdue University, United States of America

## Abstract

Endocytosis has been suggested to be crucial for the induction of plant immunity in several cases. We have previously shown that two Arabidopsis proteins, AtEHD1 and AtEHD2, are involved in endocytosis in plant systems. AtEHD2 has an inhibitory effect on endocytosis of transferrin, FM-4-64, and LeEix2. There are many works in mammalian systems detailing the importance of the various domains in EHDs but, to date, the domains of plant EHD2 that are required for its inhibitory activity on endocytosis remained unknown. In this work we demonstrate that the coiled-coil domain of EHD2 is crucial for the ability of EHD2 to inhibit endocytosis in plants, as mutant EHD2 forms lacking the coiled-coil lost the ability to inhibit endocytosis and signaling of LeEix2. The coiled-coil was also required for binding of EHD2 to the LeEix2 receptor. It is therefore probable that binding of EHD2 to the LeEix2 receptor is required for inhibition of LeEix2 internalization. We also show herein that the P-loop of EHD2 is important for EHD2 to function properly. The EH domain of AtEHD2 does not appear to be involved in inhibition of endocytosis. Moreover, AtEHD2 influences actin organization and may exert its inhibitory effect on endocytosis through actin re-distribution. The coiled-coil domain of EHD2 functions in inhibition of endocytosis, while the EH domain does not appear to be involved in inhibition of endocytosis.

## Introduction

Eukaryotic cells require endocytosis for uptake of extra-cellular substances and internalization of plasma membrane proteins for transport to endosomes [1]. Endocytosis regulates and is involved in many important processes, including several signaling pathways [2]–[4]. Recent research has revealed that plants also require endocytosis for important processes including development [5] and defense against microorganisms [6], [7]. Recent studies conducted in plant systems have elucidated possible functionalities of plant endocytic compartments and the flow of endocytosed material throughout plant cells [7]–[12].

Endocytosis depends on a large number of protein-protein interactions mediated by specific modules. One such module is the EH (Eps15 homology) domain first identified in Eps15 [13], [14]. The EH domain structure generally consists of two EF-hands and a helix-loop-helix structure that binds calcium (or a pseudo EF-hand), connected by an anti-parallel beta-sheet [15]–[17]. Thirty-five EH-containing proteins were identified so far in different species, with 11 proteins identified in human, among them EHD1-4 (EH domain containing proteins), Eps15 and Intersectin 1–2 [18].

One EHD (**E**ps15 **H**omology **D**omain) ortholog exists in Drosophila and C. elegans [19]–[21] and four orthologs are known in vertebrates. All mammalian EHDs share a similar structure: An N-terminal domain with a G-domain and nucleotide binding motif, GxxxxGKTxxxxxx (P-loop), DxxG and NKxD, two helical regions which produce a lipid binding surface and a C-terminal EH domain containing two EF Ca^2+^ binding motifs [22], [23]. A Nuclear localization signal (NLS) was also predicted for all the family members. Despite their high homology (ranging from 70% between EHD1 and EHD2 and up to 86% between EHD1 and EHD3) and similar domain structure, the mammalian EHDs (EHD1-4) differ in tissue distribution and function [22], [24]–[26]. EHD2 was localized to the plasma membrane, as well as to small intracellular tubules [22], [27], [28] and was shown to interact with phospholipids [22], [23]. EHD2 was also shown to interact with EHBP1, a possible actin binding protein, and its overexpression led to inhibition of internalization of transferrin. Its overexpression also led to actin reorganization [28]. In addition, a role for EHD2 in recycling has been suggested [29]. It was recently shown that mammalian EHD2 has a role in nucleotide dependent membrane remodeling and that its ATP binding domain is involved in dimerization, thereby creating a membrane binding region. Nucleotide binding is important for association of EHD2 with the plasma membrane, since a nucleotide free mutant (EHD2 T72A) failed to do so in cells [23].

Based on the crystal structure recently solved for mouse EHD2, it was suggested that EHD2 dimerizes and interacts with membranes via ionic interactions, possibly with the insertion of several residues into the hydrophobic phase of the lipid bilayer. It was also suggested that the EHDs be included in the Dynamin superfamily based on their G-domain structure and ability to hydrolyze ATP [23].

We recently reported the isolation and characterization of two Arabidopsis EH domain containing proteins (AtEHD1 and AtEHD2; [30] Both proteins contain an EH domain with two EF calcium binding hands, a P-loop (GxxxxGKS/T in general and in AtEHD1/2: GQYSTGKT, 100% conserved with the human EHD1 P-loop) and DxxG (DTPG in AtEHD1/2) with a predicted ATP/GTP binding site, a bipartite NLS and a coiled-coil or helical domain, as well as a Dynamin-N motif (Dynamin like GTPase domain). The two proteins were found to be involved in endocytosis in plant systems, and to possess functions similar to those of mammalian EHDs. AtEHD2 was found to have an inhibitory effect on endocytosis of both FM-4-64 in plant cells and transferrin in mammalian cells [30]. We have also demonstrated that plant EHD2 binds the cytoplasmic domain of the LeEix2 receptor and inhibits its internalization and signaling [6].

The fungal protein ethylene-inducing xylanase (EIX) [31] is a well-known protein elicitor of defense response reactions in tobacco (*Nicotiana tabacum*) and tomato (*Solanum lycopersicum*) plants [32], [33]. EIX induces ethylene biosynthesis, electrolyte leakage, expression of PR proteins and HR in specific plant species and/or varieties [33]–[36]. EIX was shown to specifically bind to the plasma membrane of both tomato and tobacco responsive cultivars [37]. The response to EIX in tobacco and tomato cultivars is controlled by a Leucine-rich-repeat receptor-like-protein (LRR-RLP) encoded by a single locus, termed LeEix [36]. LeEix2 contains the conserved endocytosis signal Yxxφ within the short cytoplasmic domain, and mutation in this endocytosis motif resulted in abolishment of HR induction in response to EIX, suggesting that endocytosis plays a key role in mediating the signal generated by EIX that leads to HR induction [36]. This was also confirmed recently when we found EIX to induce endocytosis of LeEix2 [6].

In this work we analyzed the function of different domains within the plant EHD2 protein, and we show that the ability of plant EHD2 to bind the LeEix2 receptor is mediated by the EHD2 coiled-coil. The coiled-coil of EHD2 is responsible, at least in part, for the ability of EHD2 to attenuate LeEix2 endocytosis and signaling. Truncated EHD2 lacking the coiled-coil lost most of the ability to attenuate LeEix2 signaling, while another truncation mutant lacking the EH domain retained this ability. Swapping domains between AtEHD2 and AtEHD1 (which does not inhibit LeEix2 endocytosis and signaling) leads to similar findings. Interestingly, we also found that AtEHD2 causes actin reorganization, similarly to mammalian EHD2.

## Results

### Generation and localization of AtEHD2 mutant forms

EHDs possess several domains that could potentially mediate various functions. AtEHD1 and AtEHD2 share 80% homology and essentially contain the same domains [30]. As a first step towards determining which domain of EHD2 mediates inhibition of endocytosis and EIX signaling, point mutations in the EH domain and P-loop were generated.

**Figure 1:**
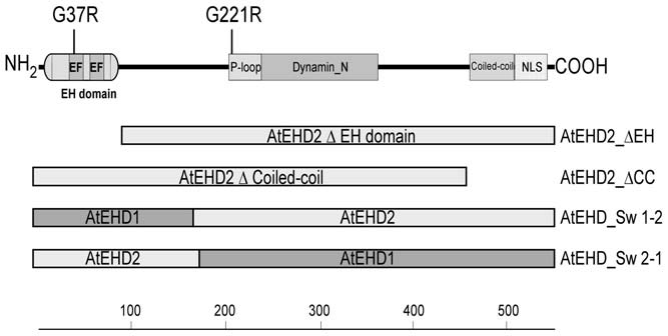
Schematic representation of AtEHD2 mutant forms. G37R = EH domain point mutation. G221 = P-loop point mutation. ΔEH = truncation mutant lacking EH domain (amino acids 62–514 of AtEHD2). ΔCC = truncation mutant lacking coiled-coil domain (amino acids 1–487 of AtEHD2). AtEHD_Sw 1–2 = swapped protein (amino acids 1–156 of AtEHD1 fused to amino acids 163–514 of AtEHD2). AtEHD_Sw 2-1 = swapped protein (amino acids 1–162 of AtEHD2 fused to amino acids 157–545 of AtEHD1).

**Figure 2:**
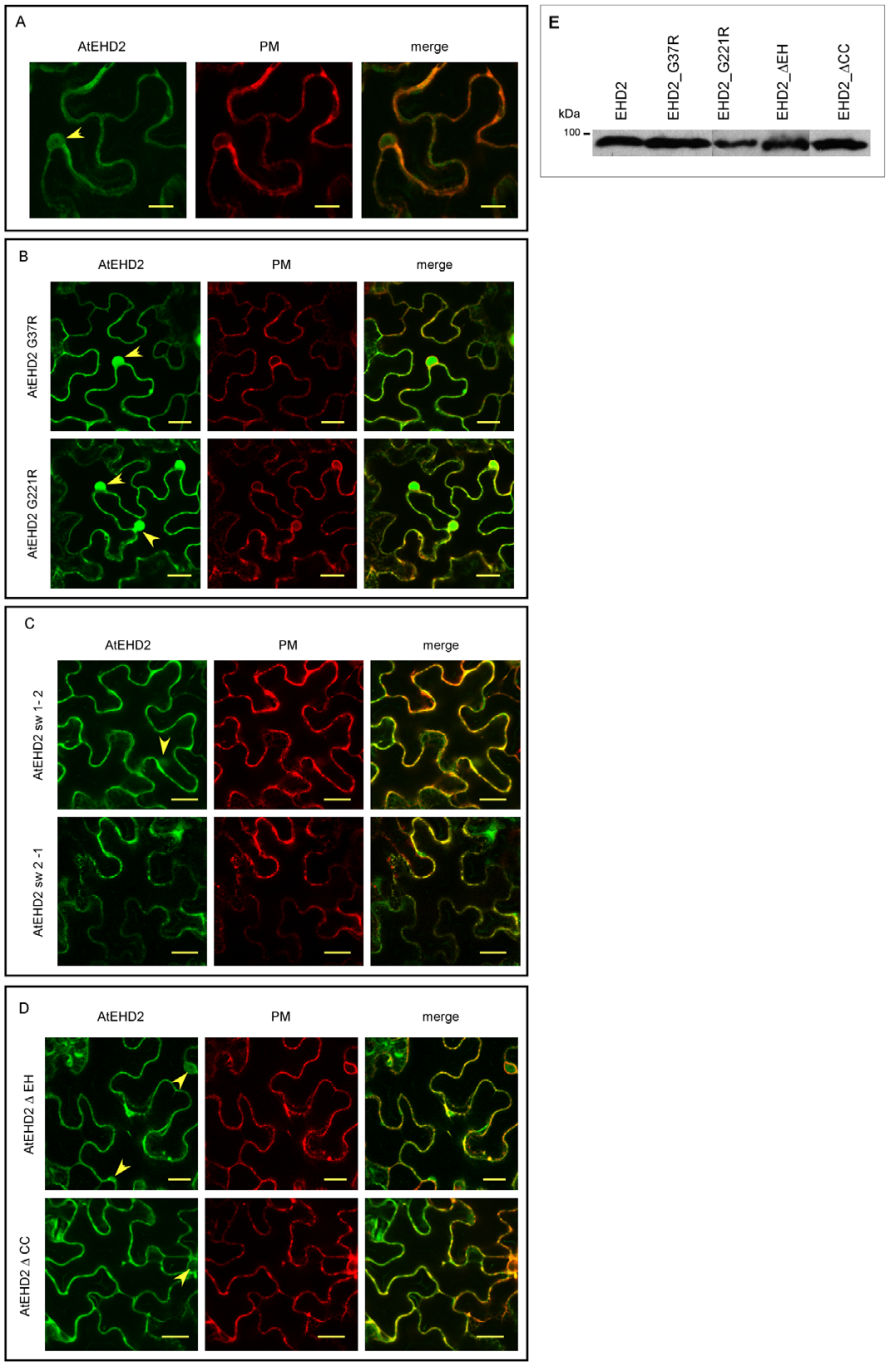
Localization of AtEHD2 and mutant forms and co-localization with a plasma membrane marker. *N. benthamiana* leaves transiently expressing PM-rk CD3-1007-cherry [39] and AtEHD2 forms as indicated, 48 hours after transformation, were visualized under a laser-scanning-meta confocal microscope (zeiss). A AtEHD2. B point mutations. C swapped proteins. D truncated proteins. Bars = 20 µm. Arrowheads indicate nuclear localization. E SDS-PAGE analysis of the expression of the various EHD2 forms. Proteins were transiently expressed in *N.benthamiana*. 48 hours after injection, total plant proteins (30 µg/lane) were extracted and subjected to 12% SDS-PAGE, transferred to a nitrocellulose membrane and probed with anti-GFP antibodies.

AtEHD2 contains an EH domain with two EF calcium binding hands and a P-loop (GQYSTGKT). Point mutations were generated in both the EH domain and the P-loop. Since the P-loop of AtEHD2 is 100% conserved with the human EHD P-loop, we chose to mutate (within the P-loop) the Glycine that generated a dominant negative mutant in human EHDs, to Argenine [38], thereby generating AtEHD2_G221R. The EH domain of plant EHDs bears lower homology to the mammalian EHDs EH domain (32% homology in the EH domain region between hEHD2 and AtEHD2) and is located at the N-terminus of the protein while the EH domain in mammalian EHDs is located at the C-terminus of the protein [30]. Though the crystal structure of EHD2 was recently solved [23], it is difficult to extrapolate from the mouse crystal to plant EHD2, since the order of the domains is shuffled. Therefore, since Glycine is known to be important in or near the EH domain of the animal and mammalian EHDs [19], [38], we examined the homology between the EH domain of all plant ESTs available, and chose to mutate the most conserved Glycine across all plant species available in the databases within the EH domain, thereby generating AtEHD2_G37R. These two point mutations are depicted schematically in Figure 1. The mutated proteins were generated as N-terminal GFP fusions under the control of the 35S promoter. Their localization is presented in Figure 2b, in conjunction with a membranal marker Pm-rk CD3-1007 (PM) [39]. The AtEHD2_G37R mutant (Figure 2b) appears to have similar localization as the wild type AtEHD2 (Figure 2a), and is co-localized with the membranal marker. AtEHD2_G37R does however stain the nucleus more strongly than wild type AtEHD2 (Figure 2ab). The AtEHD2_G221R mutant also stains the membrane to some extent. This is somewhat surprising as analogous mammalian mutations in EHD1 (G65R), and in EHD2 (T72A), are no-longer present on membranal structures [23], [38]. AtEHD2_G221R does appear to be mis-localized, as the nuclei are stained very strongly (Figure 2b). Perhaps plant EHD2 has additional elements which help tether it to the membrane, as wild-type EHD2 resides primarily in the plasma membrane [30]. The nucleotide free mammalian EHD2 mutant T72A was reported to retain the ability to bind to liposomes, despite being primarily cytosolic in localization [23].

Although AtEHD1 and AtEHD2 share 80% similarity, AtEHD1 does not inhibit endocytosis [30] or affect EIX signaling [6]. The EH domain is the least similar domain between the two proteins; therefore, we hypothesized that the EH domain might be involved in mediating inhibition of endocytosis. Therefore, in addition to point mutations we also generated “shuffled” proteins by swapping the EH domain between AtEHD1 and AtEHD2. Amino acids 1–156 of AtEHD1 were fused to amino acids 163–514 of AtEHD2 (termed AtEHD_Sw 1–2); and amino acids 1–162 of AtEHD2 were fused to amino acids 157–545 of AtEHD1 (termed AtEHD_Sw 2-1). These swaps are depicted in Figure 1. The swapped proteins were generated as N-terminal GFP fusions under the control of the 35S promoter. Both proteins retain membranal localization (Figure 2c) which is expected as both original AtEHDs are localized to the plasma membrane [30].

In addition to the point mutations and swapped proteins, we also generated truncated proteins. AtEHD2_ΔEH lacks the EH domain (Figure 1) while AtEHD2_ΔCC lacks the coiled-coil or helical domain (Figure 1). The mutated proteins were generated as N-terminal GFP fusions under the control of the 35S promoter. Both proteins were also able to retain plasma membrane localization (Figure 2d), though the AtEHD2_ΔCC deletion lost the ability to stain the nucleus (the bi-partite NLS of AtEHD2 is located at the end of the coiled-coil sequence [30]. We verified that the different mutated EHD proteins are expressed at a similar level (Figure 2e).

### Internalization of FM-4-64 in the presence of AtEHD2 mutant forms

**Figure 3:**
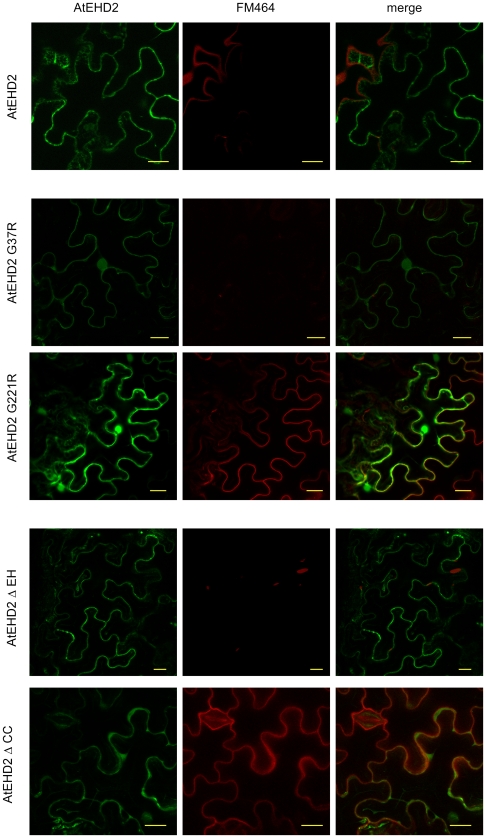
Internalization of FM-4-64 in leaf tissue expressing AtEHD2 and mutant forms. *N. benthamiana* leaves transiently expressing AtEHD2 forms as indicated were injected with 5 µM FM-4-64 48 hours after transformation. Leaf sections were visualized under a laser-scanning-meta confocal microscope (zeiss) 60 minutes after injection. Bars = 20 µm.

The styryl dye FM-4-64 was shown to enter plant cells via endocytic pathways, and is commonly used as an endocytic marker in studies conducted in plants [30], [40]–[42]. We have previously shown that AtEHD2 inhibits the internalization of FM-4-64 in plant cells [30]. We therefore tested the mutant forms of AtEHD2 described above for their ability to inhibit internalization of FM-4-64. Leaf epidermal cells of *Nicotiana benthamiana* transiently expressing one of the above described fusion proteins: AtEHD2-GFP or AtEHD2_G37R-GFP or AtEHD2_G221R-GFP or AtEHD2_ΔEH-GFP or AtEHD2_ΔCC-GFP were injected with 5 µM FM-4-64 with a needless syringe. Sixty minutes after FM-4-64 injection, leaf sections were visualized under a laser-scanning confocal microscope. As can be seen in Figure 3, the mutations AtEHD2_G221R and AtEHD2_ΔCC both lose the ability to inhibit FM-4-64 internalization. Mutants AtEHD2_G37R or AtEHD2_ΔEH retain the ability to inhibit FM-4-64 internalization. These results provided a clue that the EH domain may not be responsible for endocytosis inhibition, despite the fact that the EH domain shows the lowest homology between AtEHD1 (that does not inhibit endocytosis) and AtEHD2 (that inhibits endocytosis [6], [30].

### Interaction of mutated forms of AtEHD2 with LeEix2

**Figure 4:**
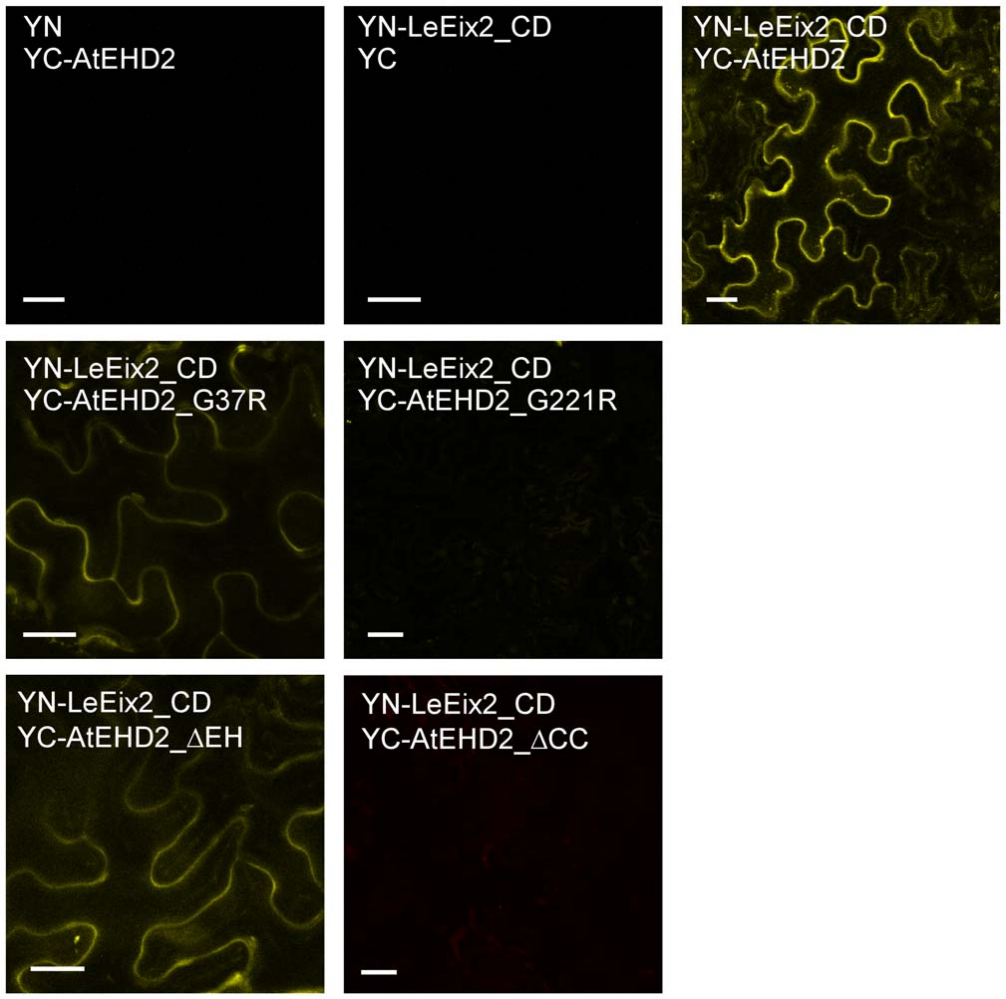
BiFc visualization of the interaction between LeEix2 and AtEHD2/mutant forms. *N. benthamiana* leaves transiently expressing YN-LeEix2_CD and YC-AtEHD2 forms as indicated. Leaf sections were visualized 48 h after transformation under a laser-scanning-meta confocal microscope (zeiss). Bars = 20 µm.

We have previously demonstrated that AtEHD2 interacts with the cytoplasmic domain of LeEix2 *in planta* in the BiFc system [6]. Here we examined reconstitution of YFP fluorescence by transient co-expression of the mutated forms of AtEHD2 and the cytoplasmic domain of LeEix2 (LeEix2_CD) in *N. benthamiana* leaves. Figure 4 shows that cells co-expressing YN-LeEix2_CD and YC-AtEHD2_G37R or YC-AtEHD2_ΔEH showed clear YFP fluorescence localized to the cell membrane. However, cells co-expressing YN-LeEix2_CD and YC-AtEHD2_G221R or YC-AtEHD2_ΔCC did not exhibit re-constitution of YFP fluorescence. YN-LeEix2_CD and all forms of AtEHD2 were examined for fluorescence with the complementary half of the YFP protein and the results were negative (Figure 4 and [6]). Our results demonstrate that the EH domain is not required for LeEix2 binding, while the coiled-coil domain is. The inability of AtEHD2_G221R to interact with LeEix2 could be attributed to the P-loop being required for this interaction – possibly in connection with membrane tethering or nucleotide binding.

### Endocytosis of LeEix2 in response to EIX treatment in the presence of mutant forms of AtEHD2

**Figure 5:**
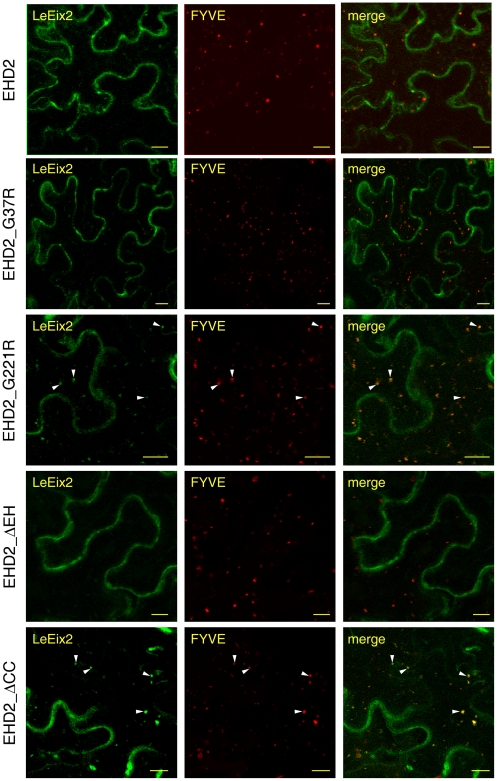
GFP-LeEix2 internalization 15 minutes after EIX application on FYVE endosomes in the presence of AtEHD2 mutant forms. *N. benthamiana* transiently expressing LeEix2 and AtEHD2-HA forms as indicated were treated with EIX (2.5 µg/gr tissue) by petiole application, and visualized 15 minutes after treatment. Bars = 20 µm.

We have demonstrated that LeEix2 undergoes endocytosis in response to EIX treatment, and that overexpression of EHD2 inhibits this endocytosis [6]. We examined whether the mutated forms of EHD2 described above retained the ability to inhibit LeEix2 endocytosis in response to EIX treatment. As can be seen in Figure 5, while AtEHD2_G37R and AtEHD2_ΔEH retained the ability to inhibit LeEix2 endocytosis in response to EIX treatment (the FYVE-expressing endosomes remained red, Figure 5), AtEHD2_G221R AtEHD2_ΔCC have lost this inhibitory activity (The FYVE endosomes are yellow indicating the presence of GFP-LeEix2 on them, Figure 5).

### EIX signaling in the presence of mutated forms of AtEHD2

**Figure 6:**
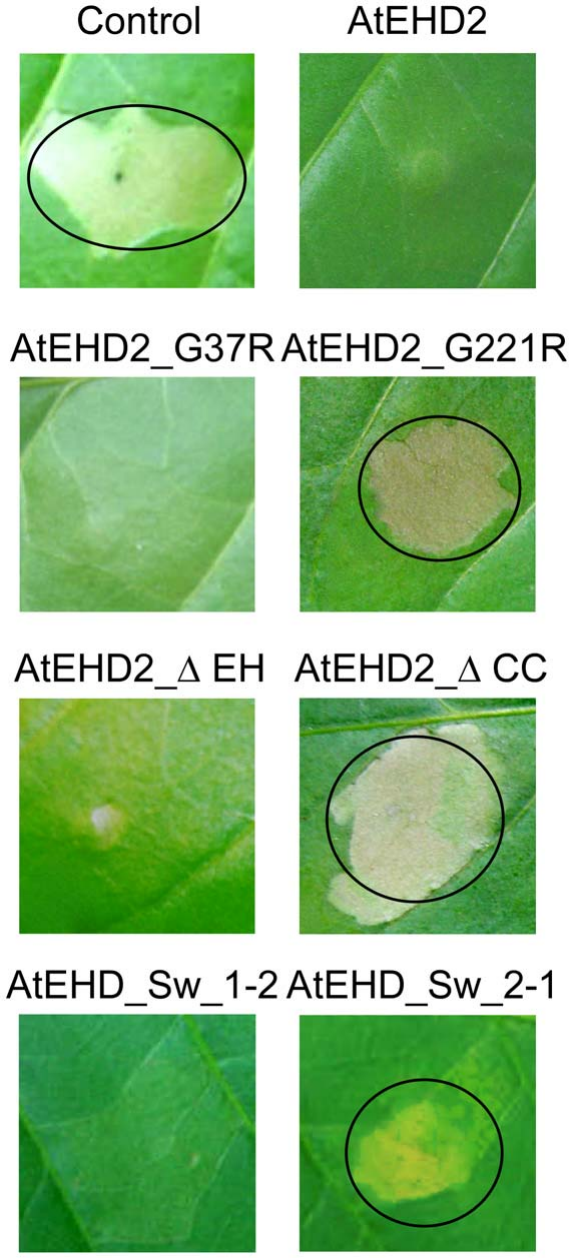
Effect of over-expression of different AtEHD2 forms on EIX-induced HR. *N. tabacum* transiently transformed with a mixture of tvEIX and AtEHD2 forms as indicated. Induction of HR was monitored 48–96 h after transformation.

We have demonstrated that AtEHD2 inhibits EIX induced cell death (HR), and induction of ethylene biosynthesis, as well as other downstream defense responses [6]. To investigate this further, we examined the effect of over-expression of mutated forms of AtEHD2 on the induction of HR and ethylene biosynthesis by EIX. Leaves infiltrated with a mixture of *Pro_35S_:tvEix* and *Pro_35S_:AtEHD2-GFP* or *Pro_35S_:AtEHD2_G37R-GFP* or *Pro_35S_:AtEHD2_*Δ*EH-GFP* or *Pro_35S_:AtEHD_Sw 1-2-GFP* exhibited no HR (Figure 6), while leaves infiltrated with a mixture either of *Pro_35S_:tvEix* and *Pro_35S_:GFP* (control) or *Pro_35S_:AtEHD2_G221R-GFP* or *Pro_35S_:AtEHD2_*Δ*CC-GFP* or *Pro_35S_:AtEHD_Sw 2-1-GFP* developed HR within 48 hours (Figure 6). The inhibition of HR induction was usually complete, though occasionally HR did appear in the AtEHD2_G37R or AtEHD2*_*ΔEH over-expression sample much later and only on part of the injected area.

**Figure 7:**
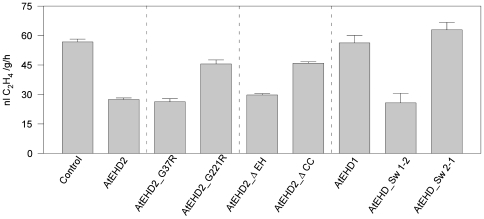
Effect of over-expression of different AtEHD2 forms on EIX-induced ethylene biosynthesis. Leaf disks of transiently transformed *N. tabacum* leaves with control (GFP) or AtEHD2 forms as indicated (48 h after transformation), were floated on a 250 mM Sorbitol solution with 2.5 µg/mL EIX (as indicated). Ethylene biosynthesis was measured after 4 hours. Error bars represent the average+SE of 4 different experiments.

To test for the effect of the mutated forms of AtEHD2 on ethylene biosynthesis, *N. tabacum* leaves were transiently transformed with *Pro_35S_:AtEHD2-GFP*, *Pro_35S_:GFP* (control), *Pro_35S_:AtEHD2_G37R-GFP*, *Pro_35S_:AtEHD2_G221R-GFP*, *Pro_35S_:AtEHD2_*Δ*EH-GFP*, *Pro_35S_:AtEHD2_*Δ*CC-GFP*, *Pro_35S_:AtEHD_Sw 1-2-GFP* or *Pro_35S_:AtEHD_Sw 2-1-GFP*. Forty-eight hours after transformation, leaf discs were prepared from the injected leaves and incubated with 2.5 µg/ml EIX. Ethylene production was measured after 4 hours of incubation. AtEHD2 greatly reduces the amount of ethylene produced in response to EIX, though it does not abolish it completely (Figure 7; [6]. AtEHD2_G37R inhibits ethylene biosynthesis to similar levels as wild type AtEHD2, while AtEHD2_G221R has partially lost this inhibitory activity (Figure 7). AtEHD2_G221R also lost the ability to inhibit HR as detailed above. Similarly, concerning the truncated proteins, AtEHD2_ΔEH retained the ability to inhibit ethylene biosynthesis while AtEHD2_ΔCC partially lost this ability (Figure 7) and completely lost the ability to inhibit HR as detailed above. It would seem that the ethylene test is more sensitive than the HR test in measuring residual levels of inhibitory activity of the mutated proteins. In connection with the “swapped” proteins, AtEHD_Sw 1-2 behaves like AtEHD2 while AtEHD_Sw 2-1 behaves like AtEHD1 (Figure 7) in the inhibition of ethylene biosynthesis, re-confirming that the EH domain is apparently not a factor in inhibitory activity on EIX signaling despite initial assumptions.

### Actin reorganization in EHD2 over-expressing cells

**Figure 8:**
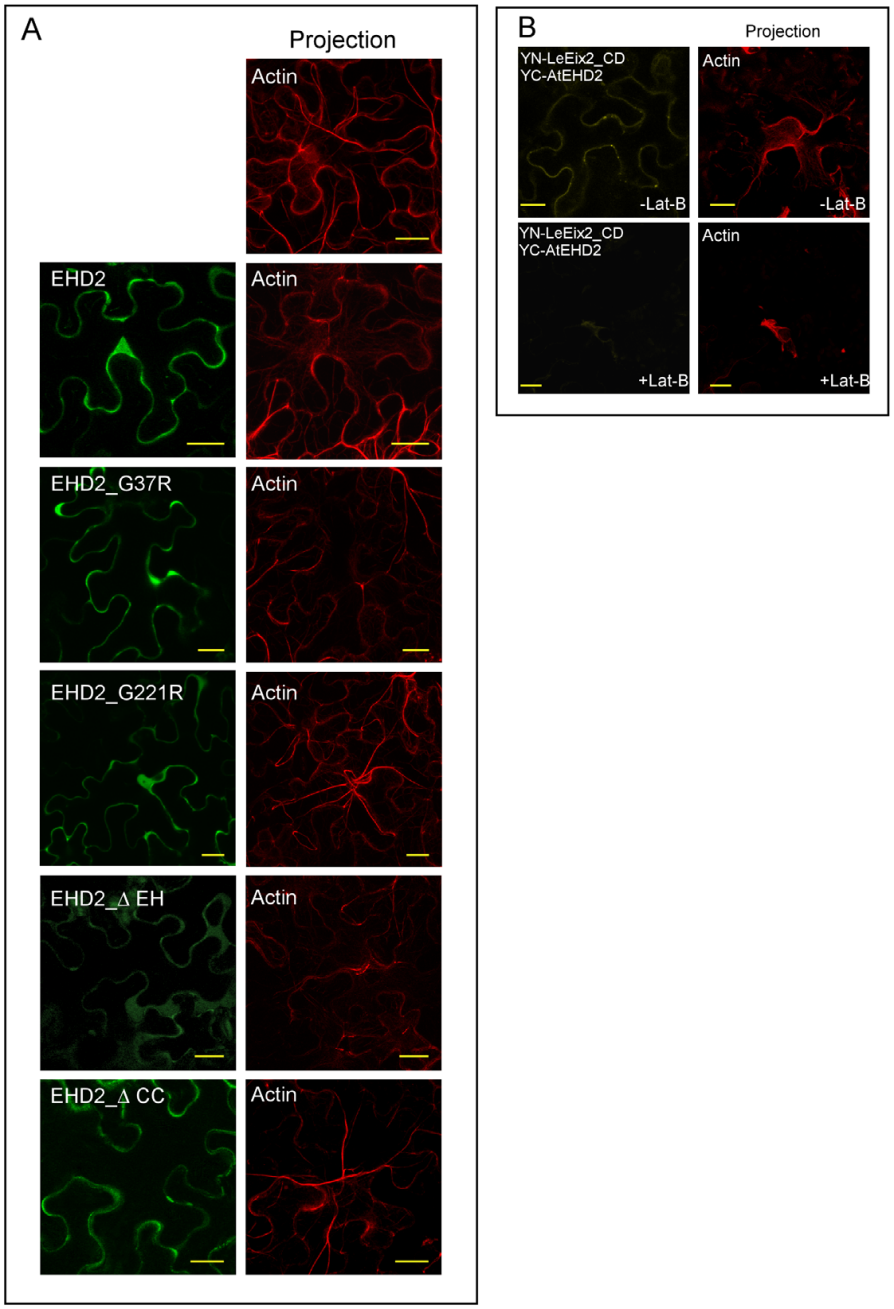
The importance of actin distribution in AtEHD2 dependent processes. (a) Effect of AtEHD2 and mutant forms over-expression on cellular actin distribution. *N. benthamiana* leaves transiently expressing the actin binding domain of Fimbrin1 (ABD)-DsRed and AtEHD2 forms as indicated, 48 hours after transformation, were visualized under a laser-scanning-meta confocal microscope (zeiss). All sections depicting actin are projections 15–20 microns in thickness. Bars = 20 µm. (b) Effect of actin disruption on the interaction between LeEix2 and AtEHD2. *N. benthamiana* leaves transiently expressing YN-LeEix2_CD and YC-AtEHD2 as well as ABD-DsRed were visualized 48 h after transformation under a laser-scanning-meta confocal microscope (zeiss), alone (top panel) or with the addition of 33 µM Latrunculin B (bottom panel). Bars = 20 µm.

It has been suggested that actin polymerization participates in early stages of endocytosis [43], [44]. EHD2 was found to cause reorganization of actin in mammalian cells upon over-expression [28]. The phenotypes observed in mammalian cells include an abundance of filamentous actin and virtual disappearance of stress actin. Figure 8 demonstrates that AtEHD2 causes a similar phenotype in *N. benthamiana* cells. Actin is marked by the expression of the actin-binding domain of fimbrin 1 [45], [46]. Note that in the AtEHD2 expressing cells the thick cable-like actin has all but disappeared, while the more gentle tendrils of mesh actin increased in abundance. We examined whether the mutated AtEHD2 forms can also influence actin distribution. As can be seen in Figure 8(a), the mutations AtEHD2_G37R and AtEHD2_ΔEH cause similar actin phenotypes as AtEHD2 upon overexpression, while the mutations AtEHD2_G221R and AtEHD2_ΔCC no longer influence actin distribution, similar to the control. Interestingly, the ability of the different forms of AtEHD2 to influence actin organization is correlated with the ability to inhibit endocytosis. Overexpression of AtEHD2, AtEHD2_G37R and EHD2_ΔEH causes actin reorganization, while overexpression of AtEHD2_G221R and AtEHD2_ΔCC does not cause actin reorganization. This could indicate that AtEHD2 inhibits endocytosis via its effect on cellular actin content.

We further examined whether the interaction between AtEHD2 and LeEix2 is actin-dependent. As can be seen in Figure 8(b), the reconstitution of YFP fluorescence in the BiFc system between AtEHD2 and the cytoplasmic domain of LeEix2 is disrupted by treatment with Latrunculin B. The entire cellular actin content appears to collapse 90 minutes after incubation of detached leaves in a 33 µM Latrunculin B solution (Figure 8b). In cells in which the actin network has collapsed, the interaction between AtEHD2 and LeEix2_CD as seen in the BiFc system, was greatly reduced (Figure 8b). This could indicate that intact actin is required for the AtEHD2 LeEix2_CD interaction; the actin reorganization caused by AtEHD2 does not in itself abolish the AtEHD2 – LeEix2 interaction. Disruption of actin with Latrunculin B could also indirectly affect the interaction between EHD2 and LeEix2 via its effects on cellular and membranal integrity, though the cells maintained normal morphology throughout the experiment.

### Association of LeEIX2 and AtEHD2 with the AP-2 complex

**Figure 9:**
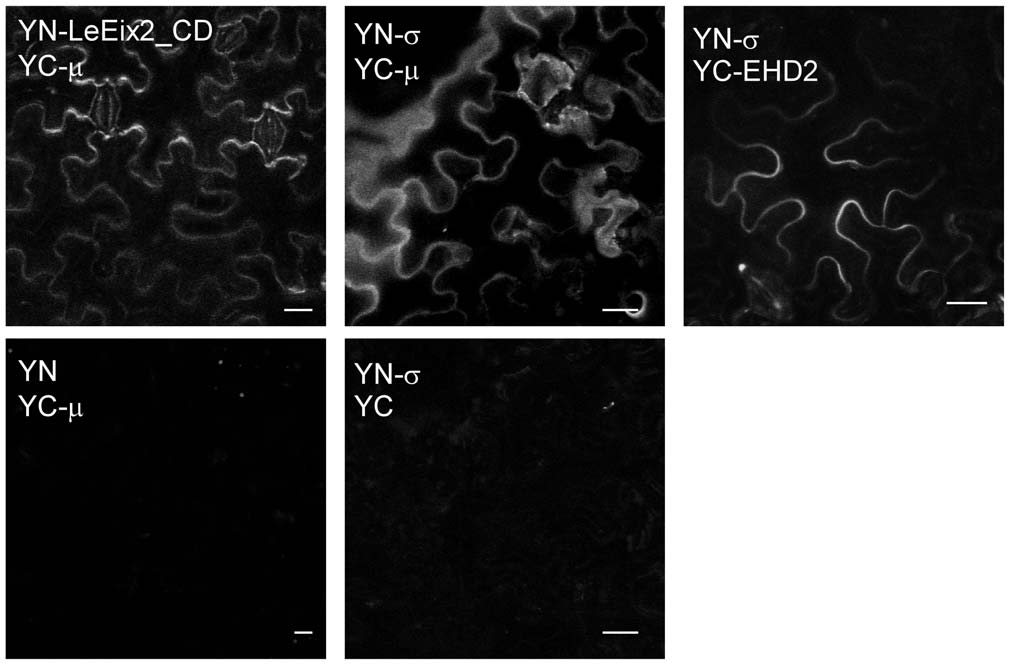
BiFc visualization of the interaction between LeEix2 and AtEHD2 via the adaptin complex. *N. benthamiana* leaves transiently expressing YN-LeEix2_CD and YC-μ-adaptin or YN-o' -adaptin (At2g19790) and YC-AtEHD2 or YN-o' -adaptin and YC-μ-adaptin as indicated. Leaf sections were visualized 48 h after transformation under a laser-scanning-meta confocal microscope (zeiss). Bars = 20 µm.

The AP2 adaptor complex works on the plasma membrane to internalize receptors and cargo molecules [47]. The μ2 chain of AP-2 was shown to interact with the YXXφ residues. LeEix2 was found to interact with AtEHD2 [6] and it contains the YXXφ signal for endocytosis. Therefore, we examined whether LeEix2 interacts with the μ2 subunit of AP-2. We examined the Arabidopsis μ subunit (accession No. At5G46630; AtAP-2 μ) reported to be involved in receptor mediated trafficking in plants [48]. The interaction between LeEix2 and the AtAP-2 μ was examined in the BiFc system. Re-constitution of YFP fluorescence by transient co-expression of the cytoplasmic domain of LeEix2 (LeEix2_CD) and AtAP-2 μ in *N. benthamiana* leaves was observed (Figure 9).

Additionally, mammalian EHD1 is known to be in complex with component proteins of the clathrin machinery such as AP-2-α-adaptin [49]. Moreover, mammalian EHD2 was shown to interact with AP-1 μ1 and Ap-2 μ2 [50]. We examined whether AtEHD2 can interact with subunits of the AP-2 complex. We first examined the interaction between AtEHD2 and At-AP-2-μ, but could not observe such interaction in the BiFc system. Therefore, we proceeded to examine the interaction between AtEHD2 and the homologous gene to the small subunit o' of the AP-2 complex (At2g19790) in the BiFc system (Figure 9).

**Figure 10:**
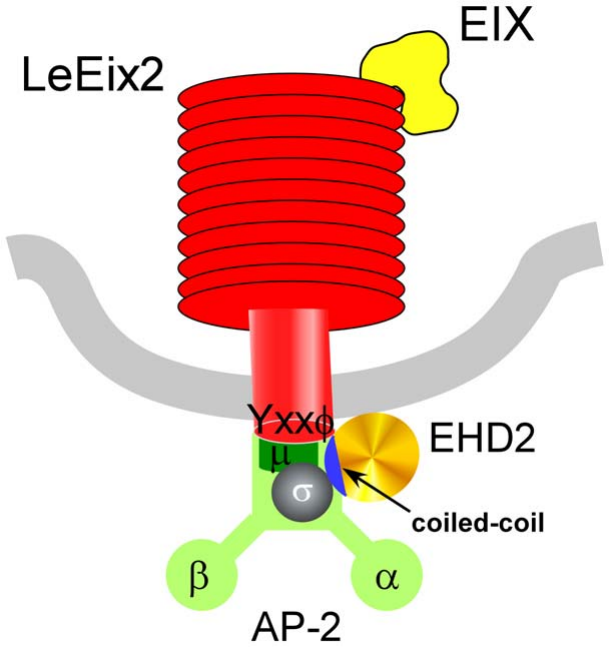
Schematic model proposing a possible conformation for the interaction between LeEix2 and EHD2. EHD2 binds o' -adaptin (AtAP-2 o' ; At2g19790) via its coiled-coil domain; LeEix2 is tethered to the adaptin complex via binding of the μ-adaptin subunit (AtAP-2 μ; At5g46630) to its YXXφ motif.

We found that LeEix2_CD interacts with AtAP-2 μ and AtEHD2 interacts with a protein that can be referred to as AtAP-2 o' . We also verified the known interaction between the two subunits of AP-2, μ and o' , in the BiFc system – see Figure 9. YN-LeEix2_CD, YC-AtEHD2 and all Adaptin proteins were examined for fluorescence with the complementary half of the YFP protein and the results were negative (Figures 4,8). Given these results, we hypothesize that AtEHD2 may bind LeEix2 via the AP-2 complex, as the diagram in Figure 10 demonstrates.

## Discussion

We recently showed that wild-type AtEHD2 is an endocytosis inhibitory protein, as reflected both in internalization of endocytosed cargo such as transferrin and FM-4-64, ligand-induced endocytosis of the LeEix2 receptor, and in signaling of the fungal elicitor EIX [6], [30]. We were also able to show that EHD2 is specific to certain endocytic systems, in particular, in internalization of receptor-like proteins possessing a YXXφ motif – as it does not inhibit the internalization or signaling of FLS2, a receptor lacking this motif [6]. Additional research conducted outside the plant kingdom has also revealed that wild type mammalian EHD2 can inhibit endocytosis [28] and has demonstrated that mammalian EHD2 can be coupled to the actin cytoskeleton. It was further shown that the nucleotide binding domain (“P-loop”) of EHD2 is important for membrane association [23]. However, to date, it was not known which domains of EHD2 are required for its inhibitory activity on endocytosis.

In this work we demonstrate that the coiled-coil or helical domain of EHD2 is crucial for the ability of EHD2 to inhibit endocytosis in plants. This domain was also required for binding of EHD2 to the LeEix2 receptor. Therefore, we suggest that binding of EHD2 to the LeEix2 receptor is required for inhibition of LeEix2 internalization. Similarly, EHD2 may bind to the transferrin receptor (which possesses a YXXφ motif) or to as of yet unknown proteins which mediate FM-4-64 internalization. We also show hereinabove that the P-loop of EHD2 is important for EHD2 to function properly, as evidenced by the loss of the ability to inhibit EIX-induced HR and to bind LeEix2 in the AtEHD2_G221R mutant. Interestingly, this mutant did retain some activity in the inhibition of EIX induced ethylene biosynthesis. This could be due to the fact that some of the protein was still localized to the plasma membrane – though its association to the membrane was undoubtedly compromised, as is evident from all other parameters examined. Ethylene biosynthesis appeared to be the most sensitive assay for AtEHD2 activity, and the mutant proteins which lost functionality in the other assays employed herein still retained some ability to inhibit ethylene biosynthesis. Our observations together with the published importance of the P-loop in mammalian EHDs leads us to the possibility that the P-loop is required for proper membranal localization of AtEHD2, while the coiled-coil in fact mediates the binding to “target” proteins thereby enabling the inhibitory function on endocytosis. Neither the P-loop mutant (G221R) nor the coiled-coil deletion (ΔCC) were able to bind the LeEix2 receptor, and both mutants lost the ability to inhibit HR. The results obtained with FM-4-64 internalization were similar – the mutants which lost the ability to bind LeEix2 and inhibit HR/ethylene biosynthesis also lost the ability to attenuate FM-4-64 internalization, as demonstrated above.

EHD2 seems to greatly influence the actin distribution within the plant cell upon overexpression; a similar phenotype was also demonstrated in mammalian cells [28], [51]. Our results could indicate that EHD2 exerts its inhibitory effect on endocytosis through the actin cytoskeleton, though more work is needed to substantiate this hypothesis. Another possibility is that inhibition of endocytosis in some way causes actin to reorganize. Further still, EHD2 may cause both phenomena in parallel. It is however evident that the ability of different forms of EHD2 to inhibit endocytosis is correlated with their ability to influence actin organization.

Interestingly, the EH domain of AtEHD2 does not appear to be involved in inhibition of endocytosis. Both the point mutation in the EH domain (G37R) and a complete deletion of this domain (ΔEH) did not affect the inhibition of endocytosis, as these mutants retained wild-type level activity. Further, swapping the EH domain between AtEHD1 (which does not inhibit endocytosis) and AtEHD2 had no effect, and the protein with the EH domain of AtEHD1 and the other domains of AtEHD2 behaved as wild type AtEHD2. In mammalians, as in plants, EHD2 is localized primarily to the plasma membrane. Interestingly, a truncation mutant of mammalian EHD2 lacking the EH domain was shown to have localization similar to that of wild type EHD2 [22]. Additionally, the truncation mutant of EHD2 lacking the EH domain was able to inhibit internalization of transferrin in a manner similar to that of wild type EHD2 [28], [51]. EHD1 was found to be important for the recycling of transmembrane cargo internalized in both clathrin dependent and independent pathways [52]. Point mutations in the EH domain of EHD1 caused effects including dominant negative inhibition of endocytosis and delayed transferrin recycling (similar to the phenotype of knock-out EHD1 mice [26]), although the mutant was only mildly mis-localized [38]. This would seem to indicate that although the EHDs share a high level of homology and similar structure/domains, both within the EHD family in mammalians and within the EHD family in plants, the fact that each EHD possesses different functionality could be related to the different domains present in the protein, whereby each function is exerted primarily through a different domain, the result being that different domains have varying importance in different EHD proteins. The EH domain, which appears to be very important in EHD1, may not be crucial for function in EHD2. Whether the EH domain of EHD2 can confer activity similar to that of EHD1 if necessary is not known, though EHD2 has been reported to regulate the exit of vesicular cargo from the ERC, a function similar to that reported for EHD1 in one case [27]. EHDs may share redundant functions in mammalians, as EHD1 knock-out mice have only a mild attenuated re-cycling phenotype [26]. Our research as well as the available data from mammalian systems seems to indicate that mammalian EHD1 and EHD3 (as well as plant EHD1) have similar or related functions, while EHD2 possesses different or at least additional roles in endocytosis. Interestingly, EHD2 is the least similar of all 4 mammalian – and plant - EHDs [30], [53].

Figure 10 is a possible model of LeEix2 – AtEHD2 binding upon EIX application (we recently demonstrated that the full LeEix2 receptor binds AtEHD2 only upon EIX application [6]). We suggest that upon EIX binding, μ-adaptin binds to the YXXφ motif within the cytoplasmic domain of the LeEix2 receptor. The AP-2 complex is assembled, and AtEHD2 binds the o' -subunit of AP-2 and/or the LeEix2 receptor directly via the coiled-coil domain. Whether direct or indirect, the interaction between EHD2 and LeEix2 is in close enough proximity that the two proteins [on their own] generate a reconstitution of YFP fluorescence in the BiFc system. Tethering of this complex to the actin cytoskeleton via additional proteins, as was reported for EHD2 in mammalians [28] may play a part in the inhibition of endocytosis, particularly given the actin reorganization phenotype that EHD2 causes upon overexpression. This is one possible model based on our results presented above; other possibilities no doubt exist, and the binding of AtEHD2 to AP-2 and/or LeEix2 will be examined further in order to elucidate the activity of different protein complexes in LeEix2 internalization and function.

## Materials and Methods

### Plant material and growth conditions

*Nicotiana tabacum* cv *Samsun* and *Nicotiana benthamiana* were grown from seeds under greenhouse conditions.

### Construction of expression vectors

*AtEHD2* was cloned in the sense orientation upstream of the *GFP* gene into the binary vector pBINPLUS [54] between the 35S-Ω promoter containing the translation enhancer signal and the Nos terminator, generating *Pro_35S_:AtEHD2-GFP*. Primers used to clone AtEHD2 are disclosed in [30]. The point-mutations of AtEHD2 were generated using site directed mutagenesis with the following primers: EHD2_G37R FOR: ggagatggtcgtgtttctagaaacgatgctacaaagttcttcgc; REV: gcgaagaactttgatgcatcgtttctagaaacacgaccatctcc; EHD2_G221 FOR: gccaaaccaatggtaatgcttctgcgccaatattccaccgg; REV: ccggtggaatattggcgcagaagcattaccattggtttggc. The truncation mutants were generated by amplifying fragments of the cDNA as desired, with the following primers: EHD2_ΔEH FOR: ggtctagaatggattcaaagcggcaag; EHD2 ΔCC REV: ggtctagacatttccttcttaaggtg. The swapped proteins were generated by double-template PCR with the following primers: EHD_Sw_1-2: P2 EHD1-REV: caattg tcaccacggaggacagagaaatcttttttgaag; P3 EHD2 FOR: gatttctctgtcctccgtggtgacaattgttgatggcttg; EHD_Sw_2-1: P2 EHD2-REV: ccactatagatgttacatttacttgtggcttaataatag; P3 EHD1 FOR: gccacaagtaaatgtaacatctatagtggatggcctg. All constructs were cloned in pBINPLUS as described above for AtEHD2. The constructs were electroporated into *Agrobacterium tumefaciens* GV3101 and the bacteria used for transient expression assays. Except where indicated otherwise, constructs used herein were cloned into pBINPLUS under the 35S promoter.

### Transient transformation

Transient expression was performed as previously described [36]. Briefly, the constructs were cloned in pBINplus [54] and introduced by electroporation into *Agrobacterium tumefaciens* strain GV3101. *Agrobacteria* were grown in LB medium overnight, diluted into an induction medium (50 mM MES pH-5.6, 0.5% (w/v) glucose, 1.7 mM NaH_2_PO_4_, 20 mM NH_4_Cl, 1.2 mM MgSO_4_, 2 mM KCl, 17 µM FeSO_4_, 70 µM CaCl_2_ and 200 µM acetosyringone) and grown for an additional 6 h until OD_600_ reached 0.4–0.5. The *Agrobacterium* culture was diluted to OD_600_ = 0.05−0.2, and the suspensions were injected with a needleless syringe into the leaves of 7–8 week old tobacco plants. Leaves were observed for protein expression 24 to 72 h after injection.

### Endocytosis of FM-4-64

Staining of tobacco cells with FM-4-64 was performed as previously described [30], [55], [56]. Briefly, FM-4-64 at a concentration of 5 µM was injected into the abaxial side of *N. benthamiana* using a needless syringe. Cells were examined under a laser-scanning confocal microscope at desired time points for FM-4-64 staining.

### Bimolecular fluorescence complementation (BiFc) analysis

*LeEix2* cytoplasmic domain (forward primer: 5′ggggccttttaggctg; reverse primer 5′ctggcggccgctcagttccttagctttccc) was sub-cloned in the Spe1 site of pSY751, downstream of the N-terminal fragment of YFP (YN). *AtEHD2* and the *AtEHD2* mutants were blunt sub-cloned into pSY752 containing the C-terminal fragment of YFP (YC) [57]. The resulting plasmids, pSY751-LeEix2_CD (YN-LeEix2_CD), pSY752-AtEHD2 (YC-AtEHD2), pSY752-AtEHD2-G37R (YC-AtEHD2-G37R), pSY752-AtEHD2-G221R (YC-AtEHD2-G221R), pSY752-AtEHD2-ΔEH (YC-AtEHD2-ΔEH), pSY752-AtEHD2-ΔCC (YC-AtEHD2-ΔCC), pSY752-At5g46630 (YC-μ), pSY751- At2g19790 (YN-o' ) were used for transient expression assays in *Nicotiana benthamiana* leaves. After incubation at 24°C for 48 h, the epidermal cell layers were viewed under a confocal microscope.

### Confocal microscopy

Cells were analyzed using a Zeiss LSM-510-Meta confocal laser scanning microscope (Zeiss, Oberkochen, Germany) with the following configuration: 30 mW Argon and HeNe lasers, 458, 477, 488, 514 and 568 maximum lines respectively. All images depict single sections, except where indicated otherwise. Contrast and intensity for each image were manipulated uniformly using Adobe Photoshop and/or ImageJ software.

### Ethylene biosynthesis measurement

Ethylene biosynthesis was assayed as described in [32]. Briefly, leaf discs from transiently transformed *N.tabacum* were incubated for 4 hours in 250 mM Sorbitol and 10 mM MES pH 5.7 supplemented with 2.5 µg/ml EIX or un-supplemented. Ethylene was measured after 4 hours using a Gas Chromatograph (Varian).

### Inhibitor and reagent application

EIX 2.5 µg/ml or 2.5 µg/gr tissue was applied to solutions or petioles of detached leaves as indicated. Latrunculin B (gift from M. Ilan) was added to solutions at a final concentration of 33 µM.
